# The influence of total hysterectomy in a cervical cancer screening population: a register-based cross-sectional study

**DOI:** 10.1186/s12913-017-2371-4

**Published:** 2017-06-20

**Authors:** Mette Bach Larsen, Ellen M. Mikkelsen, Ulla Jeppesen, Hans Svanholm, Berit Andersen

**Affiliations:** 1 Department of Public Health Programs, Randers Regional Hospital, Skovlyvej 1, 8930 Randers, NØ Denmark; 2 Department of Clinical Epidemiology, Aarhus University Hospital, Olof Palmes Allé, 43-45 8200 Aarhus N, Denmark; 3 Department of Gynaecology and Obstetrics, Randers Regional Hospital, Skovlyvej 1, 8930 Randers, NØ Denmark; 4 Department of Pathology, Randers Regional Hospital, Skovlyvej 1, 8930 Randers, NØ Denmark

**Keywords:** Mass screening, Uterine cervical neoplasm, Hysterectomy, Socioeconomic factor

## Abstract

**Background:**

High coverage of a screening program is essential to program success. Many European screening programs cover only 10–80% of their target population. A possible explanation for the low coverage may be that some women in the screening population have had a total hysterectomy, thus they are not at risk of cervical cancer. The aim of this study was to identify the prevalence of hysterectomy in the target population of the Danish National Cervical Cancer Screening Program (NCCSP) and to recalculate coverage after excluding women with total hysterectomy. Furthermore, to analyze the association between hysterectomy and sociodemographic factors within the screening population.

**Methods:**

A population-based cross-sectional study using register data on all women in the target population of the NCCSP on January 12, 2012 (women born January 12, 1947, to January 12, 1986).

The total coverage included women with hysterectomy in the target population whereas the recalculated coverage was calculated excluding women with total hysterectomy. To test the differences between the total coverage and the recalculated coverage, a two-sample z-test between the proportion of covered hysterectomized women and the proportion of covered non-hysterectomised women were used. A logistic regression model adjusted for age and sociodemographic characteristics was used to analyze the association between sociodemographic factors and total hysterectomy.

**Results:**

The coverage among women aged 26–49 years and 55–64 years were 77.4% and 72.7%, respectively. The recalculated coverage was 78.2% (26–49 years) and 79.4% (55–64 years). Recalculating the coverage did not result in coverage higher than 82.7% at any age. The effect of excluding women with total hysterectomy increased with age, reaching its maximum of 8 % points for the oldest women. Women with higher socioeconomic status (higher education and higher disposable income) had lower odds of being hysterectomized compared to other women. Also, immigrants and descendants had lower odds of being hysterectomized compared to ethnic Danes.

**Conclusions:**

Excluding women with total hysterectomy only partly explained the low coverage of the NCCSP. Thus, initiatives must be made to improve acceptability of and accessibility to the NCCSP, especially in the youngest and the oldest women.

## Background

In Europe, cervical cancer is the sixth most common cancer in women [1]. However, the incidence has been decreasing in most European countries with the introduction of cervical cancer screening programs; a similar decrease is not seen in countries without organized screening programs, such as in Eastern Europe [2, 3].

High coverage of a screening program is essential to program success. However, many European systematic cervical cancer screening programs cover only 60–80% of their target populations; some as few as 10% [4, 5]. Coverage of the Danish National Cervical Cancer Screening Program (NCCSP) is 75% where the desired quality standard is >85% coverage [6].

A possible explanation for the low coverage may be that some women in the screening population have had a total hysterectomy, thus they are not at risk of cervical cancer. Hysterectomy is the most frequent major gynecologic surgical procedure, with yearly rates varying from 5.4 per 1000 women (USA) to 1.2 per 1000 women (Norway) [7]. In a 2-year period (1998–2000), more than 10,000 hysterectomies were performed in Denmark on benign indications [8] and between 2006 and 2011, 1.7 hysterectomies were performed per 1000 women [9]; this indicates that the prevalence of hysterectomy in the NCCSP target population may be substantial. Furthermore, sociodemographic status is associated with participation in the NCCSP [10]. Thus, women with low educational attainment and low income, single women, and non-ethnic Danes are less likely to participate in the NCCSP. However, little is known about the sociodemographic characteristics of women included in the target population for cervical cancer screening who have had a total hysterectomy.

The aim of this study was to identify the prevalence of hysterectomized women in the target population for cervical cancer screening (women ages 23–64 years) and to recalculate coverage of the NCCSP after excluding hysterectomized women. Furthermore, we evaluated if hysterectomy among women in the target population was associated with selected sociodemographic factors.

## Methods

### Study design

The study was a population-based cross-sectional study using data from registries.

### Setting

Screening policies vary between countries [11] but European guidelines recommend that organized cervical cancer screening programs include women before they reach age 35 years and continue at least until they reach 64 years. Depending on disease burden and resources, screening could begin earlier and/or end later than recommended. The recommended screening interval is 3–5 years, and the recommended screening method is a sample of cellular material from the uterine cervix (cervical cytology). In Denmark, cervical cancer screening was introduced locally in 1962 and non-systematically implemented in the rest of the country until reaching nationwide coverage in late 1990s. All Danish women between 23 and 49 are now offered cervical cancer screening three years after their last screening test or last invitation; women between 50 and 64 years five years after the last screening test or last invitation [6, 12]. Danish women also have the possibility to be tested opportunistically by a general practitioner or a gynecologist. All screening procedures and treatment in hospitals, e.g. hysterectomy, is free of charge in Denmark.

### Study population

Inclusion criterion was women being in the NCCSP target population on 12 January 2012. Women, who had been in the screening population for less than one entire screening round, corresponding to women <26 years on 12 January 2012, were excluded. Furthermore, as women aged 50–54 years, are in a transition period between invitations every third and every fifth year, these women were also excluded. Thus, the study population included women born from January 12, 1947, to January 12, 1957 and from January 12, 1963 to January 12, 1986.

### Data

The study population was identified from the Danish Civil Registration System, which is updated daily and holds information such as age and gender for all Danish residents [13].

Data on hysterectomized women in the study population were retrieved from the Danish National Patient Registry (NPR), which includes data on hospital contacts since 1977. Only total hysterectomy was included in this study; women with a supracervical hysterectomy were not considered as they should remain in the screening program. From 1977 to 1994, diagnoses and procedures in the NPR were classified according to the International Classification of Diseases (ICD) 8 and afterwards according to ICD10 [14]. The following procedure codes were used to identify women with total hysterectomy: 61,020, 61,040, 61,050, 61,100, 61,780, 62,300, 72,230, 72,240, and 72,650 (ICD8) and KLCD00, KLCD01, KLCD04, KLCD10, KLCD11, KLCD30, KLCD31, KLCD40, KLDC13, KLDC20, KLDC23, KLCD96, KLCD97, KLEF00B, KLEF13, and KMCA33 (ICD10).

From the Danish National Pathology Registry [15], dates of cervical cytology were retrieved for all women between July 12, 2006, and January 12, 2012. For each woman, only the most recent cervical cytology in the study period was included. Thus, women ages 26–49 years were included with their most recently performed cervical cytology during the period July 12, 2008, to January 12, 2012, and women between 55 and 64 years were included with their most recent cervical cytology between July 12, 2006, and January 12, 2012.

From Statistics Denmark [16], data on sociodemographic characteristics of the study population by the end of 2012 were obtained. Several variables were included: Educational level was defined as low (≤10 years), middle (11–15 years) and high (>15 years). Occupation was classified as 1) employed, 2) self-employed or chief executive, 3) unemployed or receiving supplementary benefits other than social welfare, 4) retired, 5) social welfare recipient and 6) other. Marital status was categorized as married/cohabiting and single. Ethnicity was categorized as Danish, immigrant and descendant (a person born to an immigrant or to a parent with foreign citizenship). Annual disposable income (income deducted taxes, interest charges) was used as an income measure. Based on tertiles and rounded off to the nearest 100 Euros, disposable annual income was categorized as low (<22,300 Euros), middle (22,300–31,500 Euros) and high (≥31,500 Euros). We linked data using the civil registration number, assigned to all Danish residents [14].

### Analyses

Coverage is defined as the proportion of women in the target population tested at least once within the recommended screening interval. The target population is defined as all women in the age group comprised by the screening program. Thus, women were defined as covered by the NCCSP if they were registered in the Danish National Pathology Registry with at least one cervical cytology within the last 3.5 years (ages 26–49 years) or 5.5 years (ages 55–64 years), allowing for a 6-month delay for cervical cytology. Coverage of the screening program was calculated as follows:

$$ Coverage=\frac{women\  with\  cervical\  cytology\  in\ \Delta t}{women\  in\  t arget\  population\  at\  t he\  end\  of\ \Delta t} $$

Where Δt is the screening interval from July 12, 2008, to January 12, 2012, for women between 26 and 49 years and from July 12, 2006, to January 12, 2012, for women between 55 and 64 years.

The recalculated coverage excluded women from the target population who had undergone total hysterectomy.

To test the differences between the total coverage and the recalculated coverage in two independent samples, a two-sample z-test of the difference between the proportion of covered hysterectomized women and the proportion of covered non-hysterectomised women were carried at each age. The threshold of significance was adjusted for the total of 34 tests (24 for ages 26–49; 10 for ages 55–64) using the Bonferroni method. Likewise, confidence intervals at each age were determined with this method of correction for multiple comparisons. Sensitivity analyses were conducted excluding immigrants and immigrants and descendants to qualify if there may be missing data on hysterectomy among women with other origin than Danish.

A logistic regression model was used to analyze the association between having had a total hysterectomy and sociodemographic factors in the two age groups: 26–49 years (3-year screening interval) and 55–64 years (5-year screening interval). These analyses were also performed adjusting for age as a continuous variable within each age group and the categorical variables ethnicity, marital status, education, occupation and disposable income. Results are presented as odds ratios (ORs) with 95% confidence intervals (CIs). Statistical analyses were conducted using STATA version 12 (STATA Copr., College Station, Tex, USA).

## Results

Among the women with a three year screening interval (26–49 years), a total of 884,489 women were identified as the target population of the NCCSP on January 12, 2012 (Table 1). Of these, 19,644 had a total hysterectomy. Thus, the prevalence of total hysterectomy in the young target population was 2.2% (Table 1).

**Table 1:** Characteristics of the study population in age groups 26–49 and 55–64 years

	26–49 years *n* (%)	55–64 years *n* (%)
Study population	884,489	353,518
Hysterectomized	19,644 (2.2)	42,179 (11.9)
Women tested within screening interval ^a^	684,133 (77.4)	257,050 (72.7)
Marital status		
Married/Cohabiting	483,401 (54.7)	235,460 (66.6)
Divorced/widowed	100,731 (11.4)	85,142 (24.1)
Single	300,357 (34.0)	32,916 (9.3)
Education		
≤ 10 years	134,952 (16.0)	107,752 (30.9)
11–15 years	611,902 (72.5)	223,740 (64.1)
> 15 years	97,637 (11.6)	17,524 (5.0)
Occupation		
Employed	653,164 (77.8)	211,680 (71.7)
Self-employed and chief executive	42,699 (5.1)	18,029 (6.1)
Unemployed/receiving benefits ^b^	65,986 (7.9)	8743 (3.0)
Retired	^c^	39,198 (13.3)
Social welfare recipients	40,119 (4.8)	3922 (1.3)
Others	37,919 (4.5)	13,854 (4.7)
Disposable income		
< 22,300€	245,513 (28.0)	120,596 (34.1)
22,300–31,500€	318,298 (36.3)	114,458 (32.4)
≥ 31,500€	312,837 (35.7)	118,203 (33.5)
Ethnicity		
Danish	757,325 (85.6)	330,545 (93.5)
Immigrant	119,130 (13.5)	22,463 (6.4)
Descendant	8034 (0.9)	510 (0.1)

^a^ Danish women aged 26–49 are recommended to be tested every third year; women aged 55–64 every fifth year

^b^ Including maternity leave, sick leave, disability pension

^c^ No data

Among the women with a five year screening interval (55–64 years), a total of 353,518 were identified as the target population of the NCCSP on January 12, 2012 and 42,179 women were hysterectomized. The prevalence of hysterectomy among the oldest age group was 11.9% (Table 1).

### Coverage of the cervical cancer screening program

In the youngest age group, 684,133 had a cervical cytology performed, leaving the total coverage in this age group to be 77.4%. Among women with a five year screening interval, 257,050 women had a cervical cytology, thus the total coverage was 72.7% (Table 1).

**Fig. 1:**
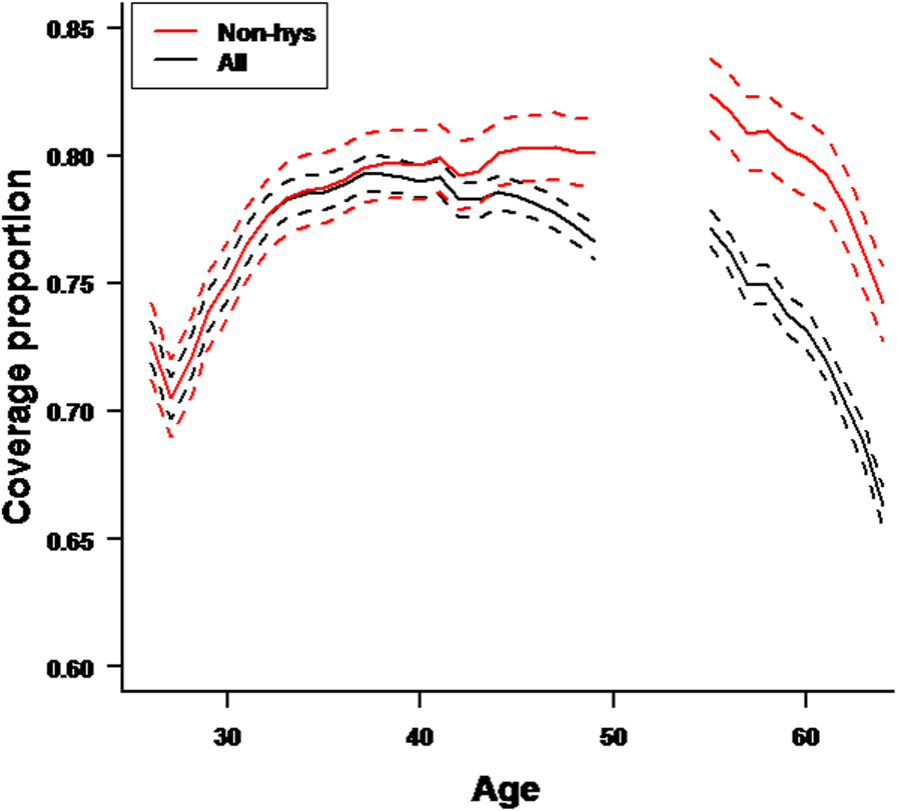
Coverage with 95% confidence intervals (*dotted lines*) of the Danish national cervical cancer screening program in different target populations (*black*: hysterectomy and non-hysterectomy, *Red*: non-hysterectomy). Note: Women aged 23–25 years are excluded from the analyses because they are not covered by a full screening interval and women 50–54 are excluded because they are in a transition period between invitations every third and every fifth year

Excluding the women with total hysterectomy, the recalculated coverage was 78.2% in the young age-group and 79.4% in the oldest age-group. As illustrated in Fig. 1, the overall pattern of coverage was similar with or without the women with total hysterectomy in the target population, both showing the lowest coverage among the youngest women and among women above 60 years.

**Fig. 2:**
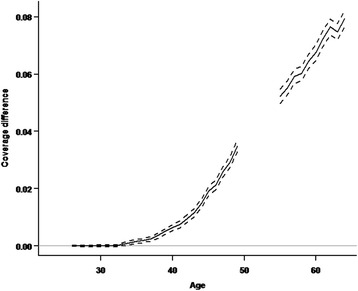
Difference in coverage of the Danish National Cervical Cancer Screening Program with and without women with total hysterectomy in the target population. Note: Women aged 23–25 years are excluded from the analyses because they are not covered by a full screening interval and women 50–54 are excluded because they are in a transition period between invitations every third and every fifth year

The effect of excluding women with total hysterectomy was statistically significant from the age 33 years. Recalculating the coverage did not result in coverage higher than 82.7% at any age (Fig. 1). As illustrated in Fig. 2, the difference between the total coverage and the recalculated coverage increased with age, reaching its maximum of 8 % points for the oldest women (Fig. 2).

Sensitivity analysis was conducted excluding first immigrants then both immigrants and descendants. Both showed a slight increase in overall coverage but the absolute difference in coverage was no altered.

### Association between hysterectomy and sociodemographic factors

In both age groups, women with higher income compared to the lowest tertile (OR_younger_: 0.72 (95% CI: 0.68–0.75) OR_older_: 0.88 (95% CI: 0.85–0.91)) and women with higher education compared to women with less than 10 years of schooling (OR_younger_: 0.35 (95% CI: 0.33–0.39) OR_older_: 0.60 (95% CI: 0.56–0.64)) were less likely to have had a total hysterectomy (Table 2). Immigrants or descendants were less likely to have had a total hysterectomy than ethnic Danes in both age groups. For women aged 26–49 years, the OR was 0.47 (95% CI: 0.44–0.51) for immigrants and 0.46 (95% CI: 0.31–0.67) for descendants. Among women aged 55–64, the ORs were 0.64 (95% CI: 0.60–0.68) and 0.76 (95% CI: 0.52–1.10), respectively. Regarding material status and occupation there was no clear association with total hysterectomy (Table 2).

**Table 2:** Unadjusted and adjusted odds ratios (ORs) with 95% confidence intervals (CIs) for association between sociodemographic factors and having had a hysterectomy in age groups 26–49 and 55–64 years

	26–49 years		55–64 years	
	Unadjusted	Adjusted ^a^	Unadjusted	Adjusted ^a^
Marital status				
Married/Cohabiting	1 (ref)	1 (ref)	1 (ref)	1 (ref)
Divorced/widowed	1.81 (1.75–1.87)	1.27 (1.22–1.32)	1.05 (1.02–1.07)	0.99 (0.97–1.02)
Single	0.45 (0.44–0.47)	0.68 (0.66–0.72)	0.62 (0.59–0.64)	0.66 (0.63–0.70)
Education				
≤ 10 years	1 (ref)	1 (ref)	1 (ref)	1 (ref)
11–15 years	0.58 (0.56–0.60)	0.70 (0.67–0.72)	0.79 (0.78–0.81)	0.89 (0.86–0.91)
> 15 years	0.20 (0.19–0.22)	0.35 (0.33–0.39)	0.44 (0.42–0.47)	0.60 (0.56–0.64)
Occupation				
Employed	1 (ref)	1 (ref)	1 (ref)	1 (ref)
Self-employed and chief executive	1.04 (0.98–1.11)	0.87 (0.81–0.93)	0.88 (0.83–0.92)	0.93 (0.88–0.98)
Unemployed/receiving benefits ^b^	0.64 (0.60–0.68)	0.96 (0.89–1.04)	0.99 (0.92–1.06)	0.97 (0.92–1.06)
Retired	^c^	^c^	1.45 (1.41–1.50)	1.13 (1.08–1.17)
Social welfare recipients	1.18 (1.10–1.25)	1.27 (1.17–1.37)	0.73 (0.65–0.82)	0.87 (0.77–0.98)
Others	0.70 (0.64–0.76)	1.00 (0.90–1.10)	0.94 (0.89–0.99)	0.92 (0.86–0.98)
Disposable income				
< 22,300€	1 (ref)	1 (ref)	1 (ref)	1 (ref)
22,300–31,500€	1.15 (1.11–1.19)	0.94 (0.89–0.98)	0.87 (0.85–0.89)	1.03 (1.00–1.07)
≥ 31,500€	1.02 (0.99–1.06)	0.72 (0.68–0.75)	0.67 (0.56–0.68)	0.88 (0.85–0.91)
Ethnicity				
Danish	1 (ref)	1 (ref)	1 (ref)	1 (ref)
Immigrant	0.41 (0.39–0.44)	0.47 (0.44–0.51)	0.57 (0.54–0.60)	0.64 (0.60–0-68)
Descendant	0.19 (0.13–0.26)	0.46 (0.31–0.67)	0.66 (0.48–0.90)	0.76 (0.52–1.10)

^a^ Adjusted for age, marital status, education, occupation, income and ethnicity

^b^ Including maternity leave, sick leave, disability pension

^c^ No observations

## Discussion

This study elaborated on the significance of total hysterectomy in a cervical cancer screening population. As expected, excluding women with total hysterectomy from the target population had the greatest effect on coverage among the older women for whom the coverage increased from 72.7 to 79.4%. Even though the effect of excluding women with total hysterectomy increased with age, recalculating the coverage did not result in coverage higher than 82.7% at any age. In addition, our study showed that within the target population for cervical cancer screening, social and cultural differences were associated with having had a total hysterectomy. Women with higher socioeconomic status (higher education and higher disposable income) had lower odds of being hysterectomized compared to other women. Also, immigrants and descendants had lower odds of being hysterectomized compared to ethnic Danes.

A major strength of this study was the register-based design, minimizing the risk of both selection and information bias. Women with a total hysterectomy were identified according to the NPR. The validity of total hysterectomies reported in NPR was studied in 1998–2000, showing a 99.8% agreement between medical records and the NPR [8]. To reach that level, coding practice has become increasingly better since the establishment of the NPR in 1977. Thus, we may not have captured all hysterectomies, especially among the elderly women. In addition, the oldest women in the target population of this study were 31 years when the NPR was established; accordingly we have not included hysterectomies performed before 1977. Thus, our estimates of total hysterectomy must be considered as minimum estimates, especially among the older women. The number of reported hysterectomies among immigrant may be underestimated since we have no data on hysterectomies in their native countries. However, the prevalence of total hysterectomy among immigrants is similar to that of descendants, who have lived their whole life in Denmark. Further, sensitivity analyses showed that it did not alter the difference in coverage when either immigrants or immigrants and descendants were excluded. Data from the Danish Pathology Register have been proven to be valid [15], thus minimizing misclassification in relation to screening coverage. Finally, data on socioeconomic characteristics retrieved from Statistics Denmark are all high quality variables with very few missing values (ranging from zero on ethnicity to 9% on occupation in our data).

Our results are in line with the Danish Glostrup Study which also showed that having a hysterectomy was associated with low sociodemographic status in the general female population [17]. A Canadian study [18] showed that the proportion of women with hysterectomy was higher among women with lower income and lower educational level.

Furthermore, coverage of the cervical cancer screening program in the Canadian study increased by 7–25% (depending on region, income, and educational level) when women with total hysterectomy were not included in the target population. Our results indicate a smaller effect of excluding hysterectomies in a Danish setting; this is consistent with a recent Danish study showing an increase in coverage from 76 to 79% after exclusion of women with total hysterectomy [19]. Nevertheless, our data support the importance of taking hysterectomy into account both when calculating the coverage of the NCCSP and when analyzing associations between sociodemographic characteristics and coverage of and participation in the NCCSP.

In the Norwegian screening program for cervical cancer, the target population is defined as women in the screening age group (23–69 years), excluding those who have had a total hysterectomy [20]. This specification could also be applied in Denmark and would give a more accurate estimate of NCCSP coverage.

## Conclusion

Excluding women with total hysterectomy from the target population significantly increased the coverage for women in the age-group 55–64 years, but did not seem to be a plausible explanation for the low coverage among the young women in the Danish NCCSP. Further, coverage among women older than 60 years also remain lower than desired. Therefore, public health interventions aimed at improving the acceptability and accessibility of the program still need to be considered.

## Abbreviations

CI: Confidence interval
NCCSP: National Cervical Cancer Screening Program
NPR: The Danish National Patient Registry
OR: Odds ratio
ICD 8/10: International Classification of Diseases version 8/10
